# Antiphospholipid Syndrome Secondary to Lupus Anticoagulant: Case Report for Clinical Anticoagulation Determination

**DOI:** 10.7759/cureus.27702

**Published:** 2022-08-05

**Authors:** Constantino G Lambroussis, Donald Foster, Amit Sharma

**Affiliations:** 1 Osteopathic Medicine, Lake Erie College of Osteopathic Medicine, Elmira, USA; 2 Family Medicine, Arnot Ogden Medical Center, Elmira, USA

**Keywords:** aps, autoimmune, anticoagulation, apl, antiphospholipid syndrome

## Abstract

Antiphospholipid syndrome (APS) is a systemic autoimmune disorder characterized by venous or arterial thrombosis and/or pregnancy morbidity in the presence of persistent laboratory evidence of antiphospholipid antibodies (APL). APS can occur as a primary condition but can also occur in the presence of systemic lupus erythematosus (SLE) or other systemic autoimmune diseases such as rheumatoid arthritis (RA) or Sjogren’s Syndrome. Our case focuses on a 21-year-old female with a history of "going numb and having no ability to speak" with a total of approximately 20 such episodes, with no known triggers for these episodes. A hypercoagulable profile was performed and indicated an elevation in lupus anticoagulant (LA), which was also positive at repeat testing after 12 weeks, meeting the criteria for APS. Oral contraceptive pills (OCP) were stopped immediately, and she was started on daily aspirin. When hematology was consulted and evaluated, the patient reported a history of possible transient ischemic attacks (TIA); however, there was no history of deep vein thrombosis (DVT), pulmonary embolism (PE), or miscarriages. Recommendations from hematology were to continue the daily aspirin but did not recommend the addition of anticoagulation therapy. Additional recommendations included avoiding risk factors for thrombosis such as the use of birth control pills, smoking, and a sedentary lifestyle or obesity. Given the young age of our patient, as well as multiple TIA associated with APS secondary to LA, the patient was started on anticoagulation contrary to hematology’s recommendations.

## Introduction

Antiphospholipid syndrome (APS) is an autoimmune disorder where the body recognizes certain normal blood proteins that bind to phospholipids, especially b2-glycoprotein I (B2-GPI), and prothrombin, and makes antibodies against them, leading to irregular blood clotting [1]. The chances of developing symptoms increase if the patient is immobile, pregnant, has had recent surgery, smokes, has hyperlipidemia, or uses oral contraceptive pills (OCPs) or estrogen therapy [2]. Additional risk factors include a history of cancer or kidney disease [3]. APS affects about 1-5% of the population. Approximately 1/3 of strokes that occur in people younger than 50 years old are due to APS. 75-90% of people affected by APS are women. 40-50% of people with lupus also have APS [1]. APS accounts for 15-20% of all episodes of deep vein thrombosis (DVT) and 10-15% of recurrent fetal loss cases [4].

Catastrophic APS is the most severe form of APS, affecting less than 1% of APS patients. In this severe form, multiple blood clots form in small, medium, and large blood vessels in a short time period, typically within a week [5]. This may lead to multiple organ dysfunction and necessitate intensive care unit (ICU) management. The most commonly affected organs are the lungs, kidneys, brain, skin, and heart [5]. Other sites where APS can occur include the esophagus and ovaries. There is a possibility that those afflicted will develop blood cell abnormalities like thrombocytopenia [5].

There is a proposed continuum due to pathological and clinical overlap between thrombotic thrombocytopenic purpura (TTP), hemolytic uremic syndrome (HUS), as well as hemolysis elevated liver enzymes and low platelets syndrome (HELLP syndrome) with catastrophic APS [6]. There are also similarities between sepsis, diffuse intravascular coagulation, and heparin-induced thrombocytopenia [7]. APS is also a known cause of recurrent miscarriages [8]. Thrombosis can affect vessels of varying caliber in any organ, which can lead to numerous clinical manifestations [4]. Choreic movements are a rare manifestation of APS. However, the etiology is not well understood and is hypothesized to be secondary to the neurotoxic effects of antiphospholipid antibodies (aPL) impairing basal ganglia function and causing neuroinflammation. The chorea can present in a patient as focal, unilateral, or generalized [9].

The presence of aPL, anticardiolipin (aCL) immunoglobulin G (IgG) and immunoglobulin M (IgM), and anti-beta2-glycoprotein I IgG and IgM (anti-B2-GPI) indicate increased risk for APS complications [10]. The presence of lupus anticoagulant (LA) is the most concerning risk factor for APS if present [10]. Anti-B2-GPI are thought to be responsible for thrombotic events. B2-GPI is thought to function in hemostasis regulation through the reduction of platelet aggregation and interference with von Willebrand factor (vWF) activity [9]. Additional mechanisms involved in the presence of aPL are thought to include interference with nitric oxide endothelial synthase functions; aPL-induced elevation in the expression of thromboplastin in endothelial cells and monocytes; activation of factor XI of the coagulation cascade; activation of complements C3 and C5; interference with Annexin function, as well as induction of intimal hyperplasia and vascular disease [10]. In APS, thrombosis results from a combination of the alterations in the coagulation cascade, impairment of fibrinolysis, dysfunctions to the endothelium, as well as platelet interference stemming from the presence of aPL [9].

Cognitive dysfunction is known to occur in autoimmune disorders [11]. Neurologic findings in APS are thought to occur in two phases [9,12]. In the first phase, aPL binds to endothelium in the brain, resulting in its dysfunction. This dysfunction leads to microthrombosis as well as vessel inflammation and the release of neurotoxic cytokines. The second phase involves direct binding of aPL to neural cell surfaces, particularly neural cells with dopaminergic characteristics, leading to over-activation of N-methyl-D-aspartate (NMDA) glutamate receptors [9,12].

To diagnose APS, at least one clinical and one laboratory criteria must be present per the classification criteria. The diagnostic criteria were published in 1999 and updated in 2006. Clinical criteria include confirmed venous/arterial/small vessel thrombosis or gestational morbidity [10]. Gestational morbidity can be one or more unexplained fetal deaths, one or more premature deliveries secondary to pre-eclampsia/eclampsia/placental insufficiency, or three or more unexplained consecutive spontaneous abortions before the tenth week of pregnancy. Laboratory criteria include the detection of LA two or more times at least 12 weeks apart, the detection of aCL IgG/IgM two or more times at least 12 weeks apart, or the detection of anti-B2-GPI two or more times at least 12 weeks apart [10]. Of the laboratory criteria for diagnosis of APS above, as an individual result, greater emphasis is currently given to having a positive LA [8].

## Case presentation

A 21-year-old female presented to the office with a history of "going numb and having no ability to speak." She had previously been evaluated at an emergency room outside the United States of America (USA). Neurology had also evaluated her during the ER visit, but no definitive diagnosis was determined. A total of approximately 20 such episodes have occurred. With the exception of the single emergency room visit outside the USA, she had not sought any other treatment as the episodes resolved after 30 minutes to 3 hours. She had ignored the episodes as they were not debilitating to her. With more recent episodes, she has not been able to speak; the numbness has been limited to the bilateral upper extremities; and there have been associated involuntary finger movements concerning focal chorea. She is unaware of any triggers for these episodes. She is a nonsmoker, does not use recreational drugs, does not consume alcohol in excess, and her current occupation is as a full-time college student. The first episode occurred while she was on a plane traveling outside the USA with her father. Past medical history is significant for hyperlipidemia and gluten intolerance. Her reaction to gluten is a generalized rash and stomach ache/discomfort. Family history is significant for her father having heart disease and transient ischemic attacks (TIA). Regarding the inability to speak, this was determined to be dysarthria as she reported that she was able to think of the words to say but was unable to vocalize them.

As she has had several transient neurologic episodes with differing symptoms and duration that always resolved without intervention, the differential diagnosis included migraines versus temporal/frontal lobe seizures versus TIA. A computed tomography (CT) scan of the head without contrast was performed, which showed no acute intracranial hemorrhage, no significant mass effect or large infarct, and no sinus air-fluid level or evidence for acute hydrocephalus. Magnetic resonance imaging (MRI) was considered; however, MRI was not performed secondary to insurance not authorizing the imaging study. A hypercoagulable profile was performed and indicated an elevation in LA, which was also positive at repeat testing after twelve weeks. For this testing, plasma LA was assessed utilizing the Dilute Russell Viper Venom Time (dRVvT) test as well as the Silica Clotting Time (SCT) test. Results for dRVvT indicated a ratio of 1.25 initially, and 1.20 on repeat at 12 weeks. SCT results showed a ratio of 1.09 initially and 0.95 upon repeat at 12 weeks. With the elevated LA noted twice with laboratory testing 12 weeks apart via dRVvT, she met criteria for APS. She was contacted immediately and ordered to stop her OCP and was started on oral Aspirin 325 mg daily. She was additionally referred to see hematology at that time.

Hematology evaluated her due to the persistent LA noted on repeat laboratory studies 12 weeks apart. She reports a history of a possible TIA; however, she has no history of DVT/PE/miscarriages. The hematologist's recommendations were to continue taking daily aspirin but did not recommend the addition of anticoagulation therapy. Additional recommendations included avoiding risk factors for thrombosis such as the use of birth control pills, smoking, and a sedentary lifestyle or obesity. In this particular case, given the very young age of our patient, suspected focal chorea with the described involuntary finger movements, as well as multiple TIA associated with APS secondary to LA, the patient was started on anticoagulation contrary to hematology’s recommendations. Although this is contrary to current evidence-based recommendations, the overall prevention of TIA progressing into a possible future cerebral vascular accident (CVA), or the development of a DVT/PE, poses a significant risk to life as well as a significant risk to disability, which would severely impact the overall quality of life of our patient. After initiating anticoagulative therapy, she has not had any recurrent episodes.

## Discussion

Antiphospholipid syndrome (APS) is a systemic autoimmune disorder characterized by venous or arterial thrombosis and/or pregnancy morbidity in the presence of persistent laboratory evidence of antiphospholipid antibodies [13]. Certain genetic polymorphisms have been found in people with APS, which may predispose them to make antibodies that lead to thromboses [14]. When exposed to a "trigger" such as surgery, trauma, or infection, as seen in 50-60% of cases of APS, those with a genetic predisposition may be more prone to catastrophic APS. A retrospective study that followed catastrophic APS patients for up to six years found that about 70% of the patients who survived their first event and took long-term warfarin therapy stayed blood clot free [5]. Primary prophylaxis of persistent anticoagulant-positive patients without thrombotic episodes is not currently recommended [4]. APS patients with the rare chorea presentation are at an increased risk for thrombosis and should therefore be on antiplatelet or anticoagulant therapy [9].

The diagnosis of APS must be made in order to determine if antiplatelet or anticoagulation is necessary. At least one clinical and one laboratory criterion must be present per the classification criteria. Clinical criteria include venous/arterial/small vessel thrombosis or gestational morbidity. Laboratory criteria include the detection of LA on at least two occurrences at least 12 weeks apart, detection of aCL antibody IgG/IgM on at least two occurrences at least 12 weeks apart, or the detection of anti-B2-GPI on at least two occurrences at least 12 weeks apart [10]. For the diagnosis of APS, greater emphasis is currently given to having a positive LA laboratory result [8].

## Conclusions

Patients with APS should follow up regularly with their physicians to discuss the possibility of needing other medications and precautionary measures to prevent future thrombosis. Consultation of specialists, as well as the latest available medical literature, is a common practice amongst physicians to stay current with the most recent available knowledge and make recommendations for best practices in patient care. The case study described here brings to the forefront the technical knowledge needed to make the diagnosis of APS as well as considerations regarding the therapeutics following confirmation of the diagnosis. Although there is a plethora of currently available medical information as well as recommendations for best practices, this case study brings to light an additional important point concerning practicing physicians. This is the need to be able to review recommendations, either from available literature or through the consultation of specialists, but overall still retain the autonomy to provide appropriate care on a case-by-case basis through clinical judgement. In our case, the recommendations from the hematology specialists were to not initiate anticoagulation. Upon weighing the risks and benefits of starting anticoagulation versus withholding, in our case, we determined it to be reasonable to start this therapeutic measure given the potential overall risks to life as well as the quality of life for our patient. After initiating anticoagulation therapy, our patient has not had a recurrent episode. The major consideration in our case was the numerous TIA episodes that our patient had, which involved dysarthria as well as focal chorea, along with the potential risk of a CVA, given that these numerous TIA episodes could severely impact the patient negatively.
